# Monitoring of Adverse Drug Reaction-Related Parameters in Children and Adolescents Treated With Antipsychotic Drugs in Psychiatric Outpatient Clinics

**DOI:** 10.3389/fpsyt.2021.640377

**Published:** 2021-02-25

**Authors:** Lenneke Minjon, Ivona Brozina, Toine C. G. Egberts, Eibert R. Heerdink, Els van den Ban

**Affiliations:** ^1^Division of Pharmacoepidemiology and Clinical Pharmacology, Utrecht Institute for Pharmaceutical Sciences, Faculty of Science, Utrecht University, Utrecht, Netherlands; ^2^Department of Clinical Pharmacy, University Medical Center Utrecht, Utrecht, Netherlands; ^3^Research Group Innovation of Pharmaceutical Care, University of Applied Sciences, Utrecht, Netherlands; ^4^Karakter Child and Adolescent Psychiatry, Zwolle, Netherlands

**Keywords:** drug monitoring, adverse (side) effects, antipsychotic agents, child, adolescent, psychiatry, medical record

## Abstract

**Aim:** To assess the frequency of monitoring of adverse drug reaction (ADR) related parameters in children and adolescents treated with antipsychotic drugs in psychiatric outpatient clinics and the considerations when monitoring was not performed.

**Methods:** This retrospective follow-up study included 100 randomly selected outpatients aged ≤18 years who had a first prescription of an antipsychotic drug recorded in the electronic medical records of psychiatric outpatient clinics between 2014 and 2017. They were followed for up to 3 years. This study assessed the frequency of monitoring for physical parameters (weight, height, body mass index, waist circumference, pulse, blood pressure, and an electrocardiogram) and laboratory parameters (glucose, lipids, and prolactin) before the first prescription of an antipsychotic drug as well as during its use. Monitoring frequencies were stratified by the patient characteristics (sex, age, cardiovascular risk factors, and use of other psychotropic drugs), and by location of antipsychotic drug initiation (psychiatric outpatient clinic or elsewhere). Additionally, this study assessed the considerations mentioned in the medical records for not monitoring ADR-related parameters.

**Results:** Overall, physical parameters were monitored more frequently (weight: 85.9% during the first half-year) than laboratory parameters (glucose and cholesterol: both 23.5%). There were no significant differences in monitoring at least one physical as well as in monitoring at least one laboratory parameter during the baseline period and during the total follow-up of antipsychotic drug treatment between the patient characteristics. In total, 3% of the children and adolescents were never monitored for any physical parameter, and 54% were never monitored for any laboratory parameter. For a minority of the children (14.8%) who were never monitored for laboratory parameters, considerations were recorded in their medical records, including refusal by the child or parents and monitoring performed by the general practitioner or elsewhere.

**Conclusion:** Monitoring frequencies of ADR-related parameters in children and adolescents treated with antipsychotic drugs in psychiatric outpatient clinics varied and especially monitoring of laboratory parameters was infrequent. Considerations why monitoring was not performed were rarely recorded. The optimal method of monitoring and documentation thereof should become clear to optimize the benefit-risk balance of antipsychotic drug treatment for each child.

## Introduction

Antipsychotic drugs are frequently prescribed to children and adolescents (hereafter referred to as *children*) to treat psychiatric disorders, including anxiety disorders, behavioral disorders, irritability associated with autism, tic disorders, and attention-deficit/hyperactivity disorder (ADHD) (1, 2). Prescribing is commonly off-label because the evidence for efficacy of these drugs in this young and vulnerable population is scarce (3, 4). Furthermore, it is well-documented that antipsychotic drugs frequently cause bothersome and even severe adverse drug reactions (ADRs), including cardiometabolic, endocrine, and extrapyramidal adverse effects (4, 5). Examples of these adverse effects include weight gain, hypertension, gynecomastia, and parkinsonism (4–6). These ADRs can differ in frequency and relative impact in children compared to adults (7). Children seem to be more likely to experience somnolence during antipsychotic drug treatment than adults; moreover, the extent of weight gain was found to be greater in children (8). Additionally, antipsychotic-induced hyperprolactinemia is more important in children because it may have an effect on pubertal development. ADRs can have both physical and emotional consequences and thereby negatively impact children's daily lives. Therefore next to monitoring efficacy, monitoring of ADRs is important to carefully evaluate and optimize the benefit-risk balance of antipsychotic drug treatment for each child.

The development of ADRs caused by antipsychotic drugs can be monitored through related parameters, including physical parameters (e.g., weight, height, body mass index (BMI), waist circumference, pulse, blood pressure, and heart examination) and laboratory parameters (e.g., glucose, lipids, and prolactin). Monitoring instructions of these parameters are available in clinical guidelines, and in regulatory drug product information such as the information leaflet (9–12). Despite the existing guidelines and instructions, previous studies have shown a large variability in the monitoring frequencies of ADR-related parameters, and that the overall monitoring frequencies were suboptimal (13–16). The majority of these studies used administrative databases from various settings, such as insurance companies or databases of general practitioners, but questionnaires about monitoring among prescribers have also been assessed (14, 16). In-depth assessments of the medical records of children treated with antipsychotic drugs is of added value in creating a complete overview of the total antipsychotic drug therapy of the individual child and what is actually monitored and recorded in daily clinical practice, including the considerations and choices made concerning (not) monitoring for ADR-related parameters.

The primary aim of this study was to assess the frequency of monitoring of ADR-related parameters in children treated with antipsychotic drugs in psychiatric outpatient clinics and the considerations when monitoring was not performed. The secondary aim was to compare differences in monitoring frequencies between sex, age categories, children with and without cardiovascular risk factors, children who were and were not prescribed other psychotropic drugs, and children who started the antipsychotic drug treatment within the psychiatric outpatient clinics and those who started this therapy elsewhere.

## Methods

### Setting, Study Population, and Follow-Up

This retrospective follow-up study included 100 randomly selected outpatients aged ≤18 years treated with an antipsychotic drug within Karakter, a large Dutch academic child and adolescent psychiatry organization that operates in 12 locations and offers clinical and outpatient therapy to children aged ≤18 years from across the Netherlands. Children are referred to this organization by, for example, general practitioners, for diagnosis and treatment of various psychiatric disorders, including autism spectrum disorder, ADHD, conduct disorders, depression, anxiety, compulsive disorders, eating disorders, and psychosis.

Patients were eligible for inclusion if they had a first prescription of an antipsychotic drug (ATC code N05A, excluding lithium [N05AN01]) within one of the psychiatric outpatient clinics of Karakter recorded in the electronic medical records between January 2014 and December 2017 and were prescribed an antipsychotic drug more than once. The date of this first prescription (index date) was defined as having no prescription of an antipsychotic drug recorded within the electronic medical records of these psychiatric (outpatient) clinics during the 6 months prior. Children could either have started the antipsychotic drug treatment within one of the psychiatric outpatient clinics of Karakter or elsewhere, for example in another psychiatric clinic. All included children were followed from the index date until the end of antipsychotic drug use recorded within the medical record, transfer out of practice, December 2018, or 3 years of follow-up, whichever came first. During follow-up, children could switch to another type of antipsychotic drug, and the period that a child was treated with an antipsychotic drug was considered to be continuous if the gap between the end date of one prescription and the start date of the next prescription was <3 months. The children included were never hospitalized within one of the psychiatric clinics of Karakter during follow-up.

Approval for this study was obtained from the organization's institutional review board (Karakter's committee for human research; reference number 148-18). A review by a medical ethics committee was not required because of the observational nature of the study with no involvement in the children's therapy or infringement of the psychological or physical integrity of the children. All data were recoded to secure privacy.

### Data Collection

The electronic medical records were stored within a clinical information system linked to an electronic drug prescription system, which were used by the healthcare professionals to access and update medical records. Within the clinical information system information regarding the child's psychiatric therapy could be consulted, including drug treatment, physical measurements, and the laboratory test results for blood glucose, lipids, and prolactin. The electronic drug prescription system also included information on the physical measurements weight, height, BMI, pulse, and blood pressure. Both systems were used to collect the data needed for this study.

Standard operational procedures (SOPs) and a checklist were used during data collection to ensure validity. Each SOP described the location of specific information in the electronic medical records, including patient characteristics, psychiatric and somatic diagnoses, diagnoses in family history, previous and current drug use, the (main) physician of the child, test requests, physical and laboratory test results, referrals, and the location of antipsychotic drug initiation. While collecting the data, patient numbers were recoded to ensure privacy.

Medical record review and data entry were conducted by two reviewers, and seven medical records were also reviewed by the first author to check for discrepancies. Discrepancies and ambiguities of all medical records were discussed and resolved by consensus with the first author as well as the additional co-authors.

### Outcomes

Baseline information up to 31 days before the index date (start of antipsychotic drug) was collected, as well as data in 6-month timeframes (182 days) during follow-up. We assessed whether children were monitored for each ADR-related physical and laboratory parameter at least once during the baseline period, to assess if monitoring outcomes at the start of the antipsychotic drug treatment were known, and at least once during each fixed 6-month timeframe thereafter. When the follow-up time of antipsychotic drug use did not cover the complete final 6-month timeframe, this timeframe was excluded, and follow-up was censored at the end of the previous timeframe. The physical parameters included weight, height, BMI, waist circumference, pulse, blood pressure, and an electrocardiogram (ECG) and the laboratory parameters included glucose, cholesterol, low-density lipoproteins (LDL), high-density lipoproteins (HDL), triglycerides, and prolactin, based on the available clinical guidelines regarding monitoring (9, 10). A child was considered to be monitored in a certain timeframe in case the result of the monitoring parameter was recorded in the medical record of that child.

### Determinants

Differences in monitoring frequencies of the ADR-related physical and laboratory parameters across the following patient characteristics were determined: (1) sex, (2) age categories (0–11 and 12–18 years old at the index date), (3) children with a cardiovascular risk factor at the index date and children without these risk factors, and (4) children who were prescribed other psychotropic drugs within the 6 months before, up to and including the index date, and children who were not prescribed other psychotropic drugs during this period. Additionally, differences in monitoring frequencies of the ADR-related physical and laboratory parameters were determined between children who started the antipsychotic drug treatment within the psychiatric outpatient clinics and those who started this therapy elsewhere. Cardiovascular risk factors were defined as having a recorded diagnosis of diabetes mellitus, hyperlipidemia, cardiovascular disorder, or overweight, hyperlipidemia according to the laboratory results or overweight according to the BMI measurement results. For the laboratory results, the reference values were included in the same document. The BMI measurement results were compared to the cutoff values described in a guideline for pediatricians (17).

### Considerations

Furthermore, this study assessed the considerations when monitoring of ADR-related physical and laboratory parameters was not performed during the antipsychotic drug treatment, which was defined as having no monitoring results included within the medical records.

### Data analysis

Descriptive statistics were used to determine the percentage of children monitored for each physical and laboratory parameter at least once during the baseline period and every fixed 6-month timeframe thereafter. Additionally, the percentage of children was determined who had been monitored for at least one of the physical and at least one of the laboratory parameters during the baseline period and during the total follow-up period thereafter. Monitoring frequencies were stratified by sex, age categories, cardiovascular risk factors at baseline, use of other psychotropic drugs within the 6 months before, up to and including the index date, and location of initiation of the antipsychotic drug treatment. Relative risks (RRs) and 95% confidence intervals (95% CIs) were calculated when comparing strata. Statistical analyses were performed using SPSS Statistics version 25.

## Results

There were 1,877 outpatients who received a prescription of an antipsychotic drug within one of the psychiatric outpatient clinics between 2014 and 2017, who were prescribed an antipsychotic drug more than once, and who were never hospitalized within one of these psychiatric clinics during follow-up. One hundred children were randomly selected (Table 1), including only those who were ≤18 years of age at the index date and who did not have an antipsychotic drug prescription within these psychiatric (outpatient) clinics during the 6 months prior to the index date. The majority of the included children were male (79.0%), aged 6–11 years (52.0%), were prescribed risperidone at baseline (59.0%), had the initial antipsychotic drug prescription within one of the psychiatric outpatient clinics (85.0%), and were diagnosed with an autism spectrum disorder (80.0%).

**Table 1:** Characteristics of the study population (*n* = 100).

**Characteristic**	***n***	**(%)**
**Sex**		
Females	21	(21.0)
Males	79	(79.0)
**Age at index date (years)**		
0–5	9	(9.0)
6–11	52	(52.0)
12–18	39	(39.0)
**Year of index date**		
2014	27	(27.0)
2015	29	(29.0)
2016	24	(24.0)
2017	20	(20.0)
**Total duration of follow-up (years)**^$^		
<0.5	15	(15.0)
0.5–1.0	19	(19.0)
1.0–1.5	19	(19.0)
1.5–2.0	11	(11.0)
2.0–2.5	7	(7.0)
2.5–3.0	9	(9.0)
3.0	20	(20.0)
**Antipsychotic drug prescribed (at index date)**		
Risperidone	59	(59.0)
Aripiprazole	22	(22.0)
Pipamperone	10	(10.0)
Olanzapine	4	(4.0)
Quetiapine	4	(4.0)
Haloperidol	1	(1.0)
**Initial antipsychotic drug prescription**		
Within the psychiatric clinic	85	(85.0)
Elsewhere	15	(15.0)
Psychiatric disorders (ever before index date)^*^		
Autism spectrum disorder	80	(80.0)
Attention-deficit / hyperactivity disorder	47	(47.0)
Intellectual disability	17	(17.0)
Anxiety disorder (incl. OCD, PTSD, phobia)	16	(16.0)
Mood disorder	11	(11.0)
Tic disorder	11	(11.0)
Behavioral disorder	9	(9.0)
Eating disorder	4	(4.0)
Sleeping disorder	4	(4.0)
Other	23	(23.0)
>1 psychiatric disorder (included above)	76	(76.0)
Somatic disorders/problems (ever before index date)^*^		
Genetic/congenital/metabolic	15	(15.0)
Allergies/asthma/eczema	11	(11.0)
Overweight/obesity	11	(11.0)
Gastrointestinal/incontinence	7	(7.0)
Epileptic disorder	5	(5.0)
Urinary	5	(5.0)
Fetal alcohol syndrome/neonatal abstinence syndrome	4	(4.0)
Underweight	3	(3.0)
Hyperlipidemia	2	(2.0)
Cardiovascular	1	(1.0)
Other	9	(9.0)
Psychotropic drug use (6 months before index date)^*^		
Stimulants and atomoxetine	33	(33.0)
Hypnotics/sedatives	26	(26.0)
Antidepressants	6	(6.0)
Other (clonidine and lithium)	7	(7.0)
Somatic drug use (6 months before index date)^*^		
Antihistamines	7	(7.0)
Oral inhalers and montelukast	6	(6.0)
Antiepileptic drugs	3	(3.0)
Other	21	(21.0)

*Index date: first prescription of an antipsychotic drug recorded in the electronic medical records of the psychiatric outpatient clinic*.

*OCD, obsessive-convulsive disorder; PTSD, post-traumatic stress disorder*.

$ *Total duration of follow-up (years): mean 1.6, median 1.4*.

* *Recorded in the electronic medical records of the psychiatric clinic, up to and including the index date; several children and adolescents were diagnosed with more than one disorder and used more than one drug*.

### Monitoring of Physical and Laboratory Parameters

**Figure 1:**
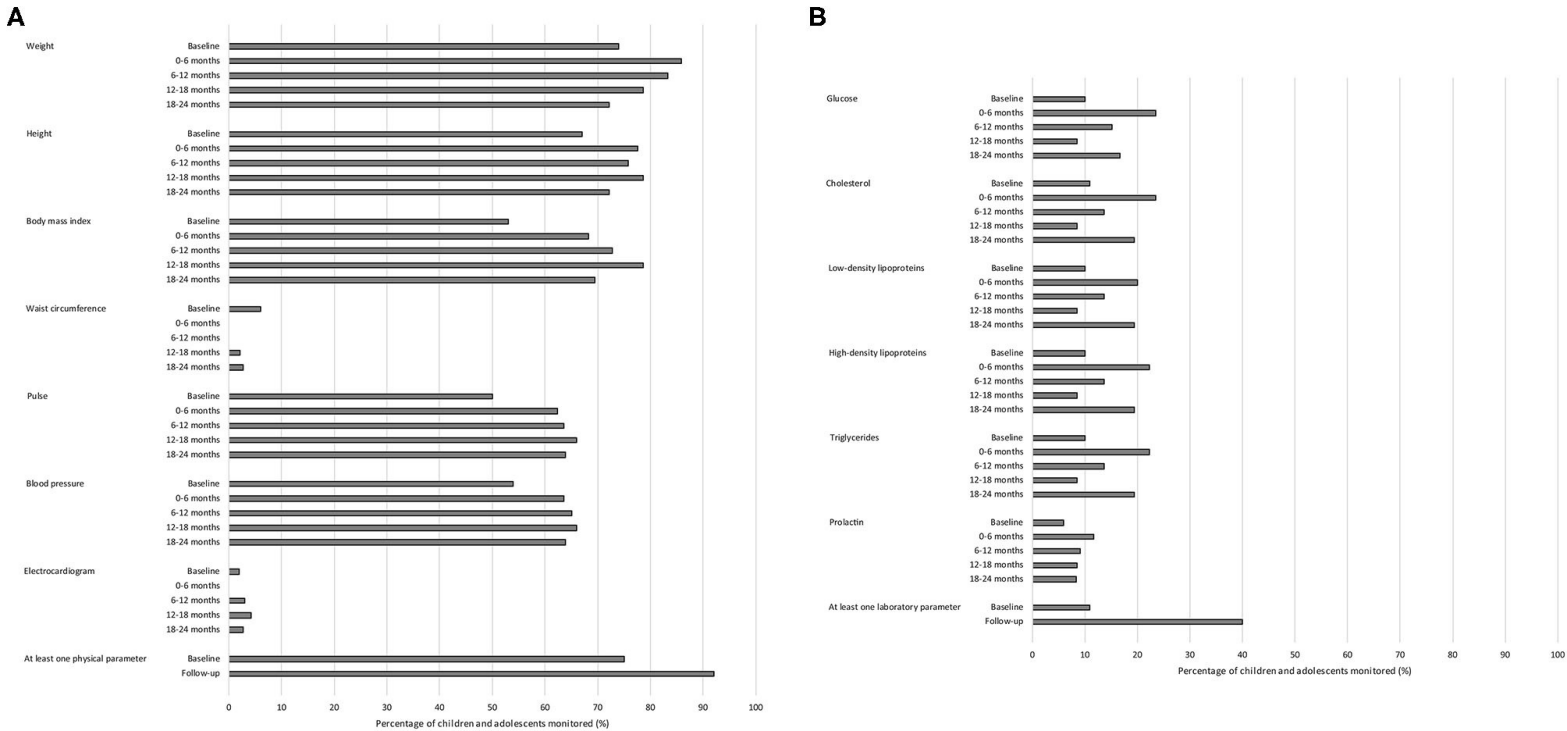
Monitoring of adverse drug reaction-related parameters in children and adolescents treated with antipsychotic drugs in psychiatric outpatient clinics. **(A)** Monitoring of physical parameters. **(B)** Monitoring of laboratory parameters. Total number of children and adolescents: baseline period *n* = 100; 0–6 months *n* = 85; 6–12 months *n* = 66; 12–18 months *n* = 47; 18–24 months *n* = 36.

Overall, physical parameters were monitored more frequently than laboratory parameters (Figures 1A,B). The physical parameter weight was monitored most frequently in children during the baseline period (74.0%) compared to the other physical and laboratory parameters. After 6 months, 85 children were still treated with an antipsychotic drug, and the physical parameters monitored most frequently in these children during this first half-year of antipsychotic drug treatment were weight (*n* = 73; 85.9%) and height (*n* = 66; 77.6%), and the laboratory parameters monitored most frequently were glucose and cholesterol (both *n* = 20; 23.5%). None of the children were monitored for waist circumference or ECG during the first half-year of treatment.

In total, 75.0% of the children were monitored at least once for one of the physical parameters during the baseline period and 92.0% during the total follow-up of antipsychotic drug treatment thereafter (Figure 1A). Additionally, 11.0% of the children were monitored at least once for one of the laboratory parameters during the baseline period and 40.0% during the total follow-up of antipsychotic drug treatment thereafter (Figure 1B). Of those children who were not monitored during the baseline period for any physical parameter (n = 25), three (12.0%) were monitored for at least one physical parameter within the first week of antipsychotic drug treatment. Of those children who were not monitored during the baseline period for any laboratory parameter (n = 89), nine (10.1%) were monitored for at least one laboratory parameter within the first week of antipsychotic drug treatment.

### Determinants

There were no significant differences in monitoring of at least one physical parameter as well as in monitoring of at least one laboratory parameter during the baseline period and during the antipsychotic drug treatment thereafter between the patient characteristics, including sex, age categories, cardiovascular risk factors at the start of antipsychotic drug treatment, and use of other psychotropic drugs within the 6 months before the start of antipsychotic drug treatment (Table 2). There were also no significant differences between children who started the antipsychotic drug treatment within the psychiatric outpatient clinics and those who started this therapy elsewhere.

**Table 2:** Monitoring of adverse drug reaction-related parameters in children and adolescents treated with antipsychotic drugs in psychiatric outpatient clinics: stratified by sex, age, cardiovascular risk factors, use of other psychotropic drugs, and location of antipsychotic drug initiation.

		**Physical parameters**				**Laboratory parameters**			
		**Baseline**		**Follow-up**		**Baseline**		**Follow-up**	
	***n***	**%**	**RR [95% CI]**	**%**	**RR [95% CI]**	**%**	**RR [95% CI]**	**%**	**RR [95% CI]**
**Sex**									
Female	21	66.7	1 (ref)	85.7	1 (ref)	9.5	1 (ref)	23.8	1 (ref)
Male	79	77.2	1.2 [0.8–1.6]	93.7	1.1 [0.9–1.3]	11.4	1.2 [0.3–5.1]	44.3	1.9 [0.8–4.2]
**Age**									
0–11 years old	61	77.0	1 (ref)	96.7	1 (ref)	16.4	1 (ref)	34.4	1 (ref)
12–18 years old	39	71.8	0.9 [0.7–1.2]	84.6	0.9 [0.8–1.0]	2.6	0.2 [0.0–1.2]	48.7	1.4 [0.9–2.3]
**Cardiovascular risk factor^#^**									
No	86	74.4	1 (ref)	91.9	1 (ref)	10.5	1 (ref)	40.7	1 (ref)
Yes	14	78.6	1.1 [0.8–1.4]	92.9	1.0 [0.9–1.2]	14.3	1.4 [0.3–5.7]	35.7	0.9 [0.4–1.9]
**Other psychotropic drugs^$^**									
No	48	72.9	1 (ref)	91.7	1 (ref)	14.6	1 (ref)	41.7	1 (ref)
Yes	52	76.9	1.1 [0.8–1.3]	92.3	1.0 [0.9–1.1]	7.7	0.5 [0.2–1.7]	38.5	0.9 [0.6–1.5]
**Initiation at the psychiatric clinic**									
No	15	60.0	1 (ref)	86.7	1 (ref)	6.7	1 (ref)	33.3	1 (ref)
Yes	85	77.6	1.3 [0.8–2.0]	92.9	1.1 [0.9–1.3]	11.8	1.8 [0.2–12.8]	41.2	1.2 [0.6–2.6]

*Children and adolescents who were monitored for at least one physical and for at least one laboratory parameter during the baseline period and during the total follow-up of antipsychotic drug treatment thereafter*.

*Baseline period: a maximum of 1 month before the first prescription of an antipsychotic drug in the psychiatric outpatient clinic, up to and including the date of this first prescription*.

# *Cardiovascular risk factors at baseline: diagnosis for overweight or overweight according to the body mass index measurements (n = 11), diagnosis for hyperlipidemia or hyperlipidemia according to the laboratory results (n = 2), diagnosis for a cardiovascular disorder (n = 1), diagnosis for diabetes mellitus (n = 0)*.

$ *Use of other psychotropic drugs within the 6 months before the first prescription of an antipsychotic drug within the psychiatric outpatient clinic, up to and including the date of the first prescription*.

*n, number of children and adolescents*.

*RR [95%CI], relative risk [95% confidence interval]*.

**Figure 2:**
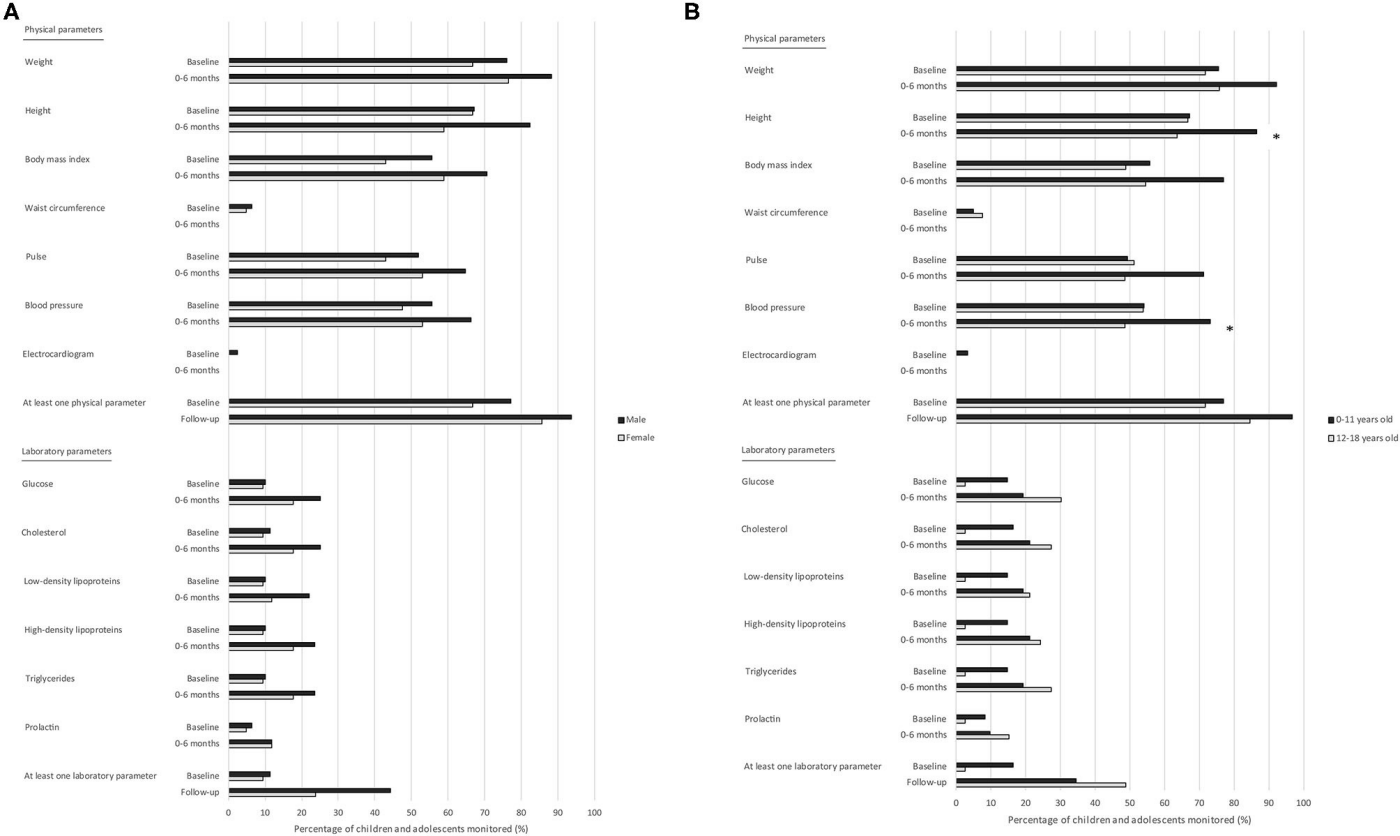
Monitoring of adverse drug reaction-related parameters in children and adolescents treated with antipsychotic drugs in psychiatric outpatient clinics: stratified by sex and age. **(A)** Stratification by sex. **(B)** Stratification by age. Total number of children and adolescents: Male: baseline period *n* = 79; 0–6 months *n* = 68; Female: baseline period *n* = 21; 0–6 months *n* = 17. Total number of children and adolescents: 0–11 years old: baseline period *n* = 61; 0–6 months *n* = 52; 12–18 years old: baseline period *n* = 39; 0–6 months *n* = 33. Baseline period: a maximum of 1 month before the first prescription of an antipsychotic drug in the psychiatric outpatient clinic, up to and including the date of this first prescription. * Significant difference; *p* < 0.05.

Assessing each physical and laboratory parameter separately, there were only few significant differences found regarding the monitoring frequency during the baseline period and during the first 6 months of antipsychotic drug treatment within one of the psychiatric outpatient clinics. There were no significant differences in monitoring between males and females (Figure 2A), but there were significant differences between the two age categories, as the physical parameters height and blood pressure were monitored relatively less frequently in children aged 12–18 years than in children aged 0–11 years (RR [95% CI]: 0.7 [0.6–1.0] and 0.7 [0.4–1.0], respectively) during the first 6 months of antipsychotic drug treatment (Figure 2B). Overall, children who were treated with other psychotropic drugs within the 6 months before the start of the antipsychotic drug treatment were monitored relatively more frequently during the baseline period and during the first 6 months thereafter for the majority of physical parameters compared to children not treated with other psychotropic drugs, but the only significant difference was found in monitoring for pulse during the baseline period (RR [95% CI]: 1.6 [1.1–2.5]). There were also no significant differences in monitoring the physical as well as the laboratory parameters when assessing only the psychotropic drugs prescribed within one of the psychiatric outpatient clinics and not elsewhere, for example by the general practitioner. Most parameters were monitored relatively more frequently when the antipsychotic drug treatment started within one of the psychiatric outpatient clinics included compared to elsewhere during the baseline period and during the first 6 months of antipsychotic drug treatment. Nevertheless, the only significant differences were monitoring for weight and waist circumference, as weight was monitored relatively more often in children who started the antipsychotic drug treatment within one of the psychiatric outpatient clinics compared to elsewhere (RR [95% CI]: 1.5 [1.0–2.3]) during the first 6 months of antipsychotic drug treatment within one of the psychiatric outpatient clinics, and waist circumference was monitored relatively less often in children who started the antipsychotic drug treatment within one of the psychiatric outpatient clinics compared to elsewhere (RR [95% CI]: 0.2 [0.0–0.8]) during the baseline period.

### Considerations

Of all included children, three were never monitored for any physical parameter during the baseline period or during the follow-up of antipsychotic drug treatment thereafter, and 54 were never monitored for any laboratory parameter. Regarding the three children who were never monitored for physical parameters, considerations why monitoring was not performed were not mentioned in their medical records. For eight of the 54 children (14.8%) who were never monitored for laboratory parameters, considerations for this lack of monitoring results were recorded in their medical records. The considerations or reasons included refusal by the child (e.g., fear of needles; *n* = 4) or parents (*n* = 1) and monitoring performed by the general practitioner or elsewhere (*n* = 4), but these results were not recorded in the medical records of the psychiatric outpatient clinic.

In the medical records of children who were monitored at least once for physical parameters during the baseline period or during the follow-up of antipsychotic drug treatment (*n* = 97), refusal by the child was mentioned in two medical records (2.1%). It was mentioned within several medical records that monitoring of physical parameters was also performed by the parents (*n* = 12; 12.4%), general practitioner (*n* = 10; 10.3%), or pediatrician (*n* = 2; 2.1%), though it was not clear if these monitoring results were always recorded in the medical records of the psychiatric outpatient clinics. In the medical records of children who were monitored at least once for laboratory parameters during the baseline period or during follow-up (*n* = 46), considerations or reasons for a delay in monitoring or a lack of results included also refusal by the child (*n* = 5; 10.9%), delay caused by the parents (*n* = 2; 4.3%), monitoring of glucose by the parents (*n* = 1; 2.2%), or monitoring performed elsewhere (*n* = 5; 10.9%), but the results were not recorded in the medical records of the psychiatric outpatient clinic.

## Discussion

Although most physical parameters were monitored more frequently than laboratory parameters in children treated with antipsychotic drugs in psychiatric outpatient clinics, the monitoring frequencies for the majority of the parameters were low. There were no significant differences in monitoring of ADR-related parameters between sex and between children with and without cardiovascular risk factors at the start of the antipsychotic drug treatment, and only a few between age categories (height and blood pressure), children who did or did not use other psychotropic drugs within the 6 months before the start of the antipsychotic drug treatment (pulse), and between the initiation of the antipsychotic drug treatment at the psychiatric outpatient clinics or elsewhere (weight and waist circumference). The considerations when there were no monitoring results included in the medical records were only occasionally reported, as, for example, this was only mentioned for 14.8% of the children who were never monitored for laboratory parameters. Considerations mentioned included refusal by the child or parents and monitoring performed by the general practitioner or elsewhere.

Although previous studies have shown differences in monitoring frequencies in children treated with antipsychotic drugs, it is clear that the monitoring frequencies were suboptimal (13, 15, 16, 18). Overall, it has been shown that the physical parameter weight was monitored more frequently in children treated with antipsychotic drugs compared to the laboratory parameters glucose and lipids, and waist circumference was monitored much less, which is in line with the results of this current study (14, 15, 19).

Some differences in monitoring frequencies across sex and age categories were indicated in this study. Although this current study showed no significant differences between sex, it seemed that boys were monitored relatively more frequently than girls. This study demonstrated significant differences between age categories (0–11 and 12–18 years) in monitoring for the physical parameters height and blood pressure, but there were no significant differences in monitoring for laboratory parameters. However, higher monitoring frequencies of laboratory parameters in older children were demonstrated in previous studies (20, 21). This result could also have been expected in the current study, as these differences in monitoring frequencies of laboratory parameters could be due to the fear of needles, which is generally more common in younger children (22).

Especially the monitoring frequencies of the laboratory parameters were low. Monitoring instructions of parameters are available in clinical guidelines, but these guidelines differ in which parameters they recommend to monitor and the frequency of monitoring (9–11, 23). Although there is no national clinical guideline in the Netherlands for monitoring of ADR-related parameters in children treated with antipsychotic drugs, the guideline of Accare, a large academic mental health organization for child and adolescent psychiatry in the northern part of the Nederlands, is widely used by other Dutch healthcare professionals and is published on the national website for child and adolescent psychiatry (https://www.kenniscentrum-kjp.nl/) (9). Strict use of this guideline varies among prescribers, also within Karakter. The low monitoring frequencies of the laboratory parameters could be due to the recommendation of this guideline to monitor the parameters glucose and lipids only at baseline and every 6 months thereafter when there are risk factors present. One of these risk factors is overweight. However, no significant differences were shown by this study between children with and without cardiovascular risk factors, including overweight. Overweight was the most reported cardiovascular risk factor within this study. The risk factors hyperlipidemia and diagnosis for a cardiovascular disorder were only reported in few medical records, and diabetes mellitus in none. This could be because these disorders are rare in children, or this information was not well-reported in the medical records and therefore missing.

Previous studies have shown suboptimal monitoring frequencies in children treated with antipsychotic drugs and low compliance to monitoring guidelines (15, 20, 24). Improvement in monitoring practices is needed, which is seen not only in children treated with antipsychotic drugs, but also the monitoring frequencies for adults treated in psychiatric outpatient clinics have been shown to be suboptimal according to the guidelines (25). Additionally, low monitoring frequencies are not only related to antipsychotic drug use, as low monitoring frequencies and poor adherence to clinical guidelines have also been demonstrated concerning other psychotropic drugs, including lithium, as well as somatic drugs (26–28).

As this study showed only minor differences in monitoring frequencies between patient characteristics, including sex, age categories, and children with and without risk factors present, and suboptimal monitoring frequencies were also shown by other studies including adults and other types of drugs, the reasons for suboptimal monitoring might be with the healthcare professionals (or the system) or children and caregivers themselves. Suboptimal monitoring by the healthcare professionals could be caused by the lack of a clear national clinical guideline, insufficient collaboration with other healthcare professionals, low confidence about monitoring, a lack of a reminder system or insufficient access to the equipment needed, for example a blood pressure machine (29). Despite the lack of a national guideline, the majority of the prescribers of antipsychotic drugs to children are aware that they should monitor for ADRs (14). However, when collaborating with other health care professionals, it is not always clear who is responsible to monitor for ADRs (29–31). As shown in this current study, children could also be monitored by the general practitioner or pediatrician, though it was not always clear which exact parameters were monitored elsewhere and if the results were recorded in the medical records of the psychiatric outpatient clinics, since the electronic systems were not linked. A gap between monitoring for ADRs and the rest of the antipsychotic drug treatment is concerning, as it could lead to poor monitoring, undetected abnormalities in ADR-related physical and laboratory parameters, and insufficient follow-up of the antipsychotic drug treatment. An electronic system for medical records could enhance the monitoring practices by more easily sharing monitoring results and defining whose responsibility it is to monitor the children (29). Electronic medical records do also facilitate as they improve the quality of outpatient clinic notes, including information about ADRs and follow-up information (32). However, documentation quality varies between healthcare professionals and type of care measure in regard to medication, drug allergies, and compliance with guidelines (33), as also seen in this current study. Electronic systems should be equipped to suit the needs of healthcare professionals in the evaluation and monitoring of ADRs in children treated with antipsychotic drugs (34). For example, this electronic system should also include a reminder system, not only to remind the healthcare professional that monitoring should be performed, but also to assess the parameters outcomes, for example laboratory parameters, on a later moment in time. Furthermore, the children and the caregivers play an important and active role in optimizing monitoring practices. Barriers related to the children of caregivers are refusal by the child, for example because of a fear of needles, as also shown in this study, or the caregivers who resist or simply forget to obtain the laboratory tests (35). Clear instructions and information tailored to the patient would improve monitoring practices (36). Additionally, it is important that the healthcare professional is aware of the barriers present and can anticipate the specific situation.

A strength of this study was that by reviewing the electronic medical records, a complete overview was gained of the total antipsychotic drug therapy of the individual child in the psychiatric outpatient clinics. Medical records review and data entry were conducted by only two reviewers, who used SOPs and the checklist to gather the information needed, which ensured that they gathered information consistently and no important files in the medical records were missed. However, this study also has some limitations. This study included a relatively small number of children in one mental healthcare institution in the Netherlands, although there were multiple locations involved. Especially the numbers when separating in different patient characteristics and the location of initiation of the antipsychotic drug treatment were small. To compare these groups was the secondary aim of the study. More research is needed to detect differences between those groups. The diagnoses (Table 1) were those reported in the medical records and we did not validate these diagnoses. However, this does not influence the results of this study as a child treated with an antipsychotic drug should be monitored regardless of the diagnosis. Fifteen children were prescribed an antipsychotic drug elsewhere before they were transferred to one of the psychiatric outpatient clinics, which could lead to a difference in documentation history compared to the children who started the antipsychotic drug treatment within the psychiatric outpatient clinics. Some children did not have 1 month of valid data available before the index date. Data collected depended on what was reported within the records, and notes could be missing, unclear, or incomplete. However, for this study also the free texts within a medical record were taken into account. Even if missing or unclear data has led to an underestimation of the monitoring frequencies, this would also deteriorate the quality and completeness of the medical records in the psychiatric outpatient clinics in daily clinical practice, and could lead to an incomplete transfer of information to other internal and external healthcare professionals.

### Clinical Implications

By monitoring children treated with antipsychotic drugs, abnormalities in ADR-related physical and laboratory parameters can come to light, and interventions can be performed to optimize the benefit-risk balance of the antipsychotic drug treatment for each child, including lowering the dosage, switching to another drug, a referral to a dietitian or consulting a pediatrician. When monitoring is suboptimal, this could cause severe risks, as abnormalities in blood glucose and a high body weight could result in the development of diabetes mellitus, and abnormalities in blood prolactin levels could lead to gynecomastia and galactorrhea (37, 38). On the other hand, when the monitoring frequency is excessive, this not only increases the healthcare costs, causes unneeded time investments and an administrative burden for the healthcare professionals, this can also impact the child's quality of life, considering the fear of needles and the constant reminder of the psychiatric disorder with which the child has been diagnosed. Further research is needed to gain knowledge about the optimal method of monitoring for ADR-related parameters in children, which should be captured in a clear national clinical guideline to prevent children from developing severe ADRs and to optimize the benefit-risk balance in the individual child.

## Conclusion

Overall, monitoring frequencies of ADR-related parameters in children treated with antipsychotic drugs in psychiatric outpatient clinics varied and especially monitoring of the laboratory parameters was low. There were no prominent differences in monitoring between patient characteristics, for example across sex and age categories. Considerations why monitoring was not performed were rarely recorded within the medical records. By gaining more knowledge concerning the optimal frequency of monitoring and the facilitators and barriers for monitoring in psychiatric outpatient clinics as well as for each child, monitoring practices could be improved. Monitoring leads to knowledge about the effects of the antipsychotic drug treatment in the individual child, which is essential to evaluate and improve the benefit-risk balance of the therapy.

## Data Availability Statement

The datasets presented in this article are not readily available because the dataset includes information from psychiatric outpatient clinics (for children and adolescents). Requests to access the datasets should be directed to Lenneke Minjon, l.minjon@uu.nl.

## Ethics Statement

The studies involving human participants were reviewed and approved by Karakter's committee for human research (institutional review board; reference number 148-18). Written informed consent from the participants' legal guardian/next of kin was not required to participate in this study in accordance with the national legislation and the institutional requirements.

## Author Contributions

Material preparation and data collection were performed by LM, IB, and EB. Analysis was performed by LM, IB, and EH. The first draft of the manuscript was written by LM. All authors commented on previous versions of the manuscript, contributed to the study conception and design, read and approved the final manuscript.

## Conflict of Interest

The authors declare that the research was conducted in the absence of any commercial or financial relationships that could be construed as a potential conflict of interest.
